# Metabolism-dependent bioaccumulation of uranium by *Rhodosporidium toruloides* isolated from the flooding water of a former uranium mine

**DOI:** 10.1371/journal.pone.0201903

**Published:** 2018-08-08

**Authors:** Ulrike Gerber, René Hübner, André Rossberg, Evelyn Krawczyk-Bärsch, Mohamed Larbi Merroun

**Affiliations:** 1 Helmholtz-Zentrum Dresden-Rossendorf, Institute of Resource Ecology, Dresden, Germany; 2 Helmholtz-Zentrum Dresden-Rossendorf, Institute of Ion Beam Physics and Materials Research, Dresden, Germany; 3 Rossendorf Beamline at ESRF - The European Synchrotron, Grenoble, France; 4 University of Granada, Department of Microbiology, Campus Fuentenueva, Granada, Spain; Universita degli Studi di Urbino Carlo Bo, ITALY

## Abstract

Remediation of former uranium mining sites represents one of the biggest challenges worldwide that have to be solved in this century. During the last years, the search of alternative strategies involving environmentally sustainable treatments has started. Bioremediation, the use of microorganisms to clean up polluted sites in the environment, is considered one the best alternative. By means of culture-dependent methods, we isolated an indigenous yeast strain, KS5 *(Rhodosporidium toruloides*), directly from the flooding water of a former uranium mining site and investigated its interactions with uranium. Our results highlight distinct adaptive mechanisms towards high uranium concentrations on the one hand, and complex interaction mechanisms on the other. The cells of the strain KS5 exhibit high a uranium tolerance, being able to grow at 6 mM, and also a high ability to accumulate this radionuclide (350 mg uranium/g dry biomass, 48 h). The removal of uranium by KS5 displays a temperature- and cell viability-dependent process, indicating that metabolic activity could be involved. By STEM (scanning transmission electron microscopy) investigations, we observed that uranium was removed by two mechanisms, active bioaccumulation and inactive biosorption. This study highlights the potential of KS5 as a representative of indigenous species within the flooding water of a former uranium mine, which may play a key role in bioremediation of uranium contaminated sites.

## Introduction

Heavy metal pollution of the environment is one of the biggest problems today due to its great impact on the surface and groundwater, and even in the catchment areas of drinking water [1]. In particular, uranium contamination is a result of former mining activities, by weathering of uranium containing minerals or by accidental release to the environment [2,3]. In Germany, the former uranium mine Königstein was one of the smallest mining sites in Eastern-Germany. Uranium was recovered from the underground sandstone by leaching with sulfuric acid [4]. The mining activity resulted in the release of about 2 million of cubic meters of acid pore water, which contained high amounts of uranium. Due to the stop of uranium mining in Germany, the underground is actually in the process of remediation and since 2001, the mine is flooded in a controlled way. Nevertheless, the flooding water still contains relatively high concentrations of uranium (8 mg/L) and other heavy metals. Additionally, the flooding water displays a low pH (around 3) as a consequence of the leaching process [5]. For this reason, the flooding water is treated by an elaborate chemical wastewater treatment plant. In contrast to other former uranium mines in Germany, Königstein is unique in the remediation effort, due to disturbances in the underground rock formation, it is not possible to flood the mine completely, otherwise nearby aquifers would be potentially contaminated with uranium. Therefore, the flooding water is pumped to the surface (pump-and-treat technique) and is treated by chemical precipitation and ion exchange [6].

Within natural environments the mobility and bioavailability of uranium depends strongly on its speciation and physicochemical form [7]. Particularly in acidic (< pH 5) and non-reducing conditions the free uranyl ion (UO_2_^2+^) predominates the uranium speciation [8]. Previous studies showed that uranium mainly occurs in the bulk solution as highly soluble UO_2_SO_4_-species within the flooding water of the former uranium mine [9]. Besides abiotic factors such as pH, redox potential, dissolved organic and inorganic ligands, and the presence of solid particulates [5,10,11], biological processes could have a significant impact on the migration of radionuclides. They can affect their mobility by a variety of interaction mechanisms, such as biosorption on functional groups of the cell-surface, bioaccumulation within the cell, biotransformation (reduction or oxidation of the radionuclide), and biomineralization [2,12–19].

Despite harsh environmental conditions generated in the mining process, such as high uranium and sulfate concentrations, and low pH, leading to acid mine drainage (AMD), microorganisms are able to survive and even display metabolic activity in these environments [20]. In addition, culture-independent studies of the microbial diversity within the flooding water of the former uranium mine Königstein revealed a diverse community of microbial life [21,22]. Therefore, microorganisms should be taken into consideration to investigate possible interaction mechanisms which could be helpful for biological approaches concerning bioremediation.

Investigations on the influence of yeast cells on actinides, like uranium are still in its infancy [23]. Much is known about the interaction of model organisms, such as *Saccharomyces cerevisiae* and *Escherichia coli*, with heavy metals and radionuclides [24–26]. It seems to be important to consider microorganisms from ecological niches, like the former uranium mining site Königstein, for the use in metal bioremediation approaches, since conventional technologies, such as chemical precipitation and ion exchange, are cost-intensive and often inefficient for metals at low concentrations [27–29]. Therefore, the present study aimed to describe the possible role of microorganisms that were isolated from their natural uranium-contaminated habitats as a potential alternative to conventional chemical remediation strategies, due to their adaptive tolerances and possible immobilization ability of radionuclides. We investigated an isolated yeast, KS5, which was identified as *Rhodosporidium toruloides*. *R*. *toruloides* (syn. *Rhodotorula gracilis*) belongs to the division of Basidiomycota and is an oleaginous yeast [30]. Species of *Rhodosporidium* were isolated from heavy metal contaminated soil in former studies. Heavy metal tolerance tests with this strain displayed high tolerances against several metals [31]. In addition to the high tolerances, *R*. *toruloides* offers many other biotechnological opportunities as an alternative yeast model compared to *S*. *cerevisiae*, which lacks several biochemical features [30]. Furthermore, we investigated the uranium removal capacity of the indigenous yeast KS5 and its tolerance to selected heavy metals. More precisely, different influencing factors on the uranium removal capacity of KS5 were examined, such as temperature, initial dry biomass and viability of the yeast cells. To obtain a detailed and closer look on the localization of the removed uranium on the cell membrane and possibly inside the cells, high-angle annular dark-field scanning transmission electron microscopy (HAADF-STEM) and energy-dispersive X-ray spectroscopy (EDXS) were performed. The structural parameters of the uranium complexes formed by the strain were studied using EXAFS (extended X-ray absorption fine structure spectroscopy) spectroscopy. In addition, we compared the metal tolerances of the isolated strain KS5 with the reference strain DSM 10134 to investigate natural adaption mechanisms against heavy metals. The results of our investigations provide new insights on the interaction of indigenous yeast cells with uranium, and consequently, a possible use of microbial cells for *in situ* bioremediation.

## Methods and materials

### Water sampling, microbial isolation and cultivation conditions

Samples (1 L) of flooding water from the former uranium mine in Königstein (50°54'51.8"N and 14°01'42.5"E, Saxony, Germany) were taken into sterile glass bottles. The water was stored at 4 °C until further processing. For isolation of aerobic fungal microorganisms, 500 μL of the flooding water was plated onto solid SDA (Sabouraud Dextrose Agar) medium (Peptone 5.0 g/L, Casein Peptone 5.0 g/L, Glucose 40.0 g/L, 15.0 g/L Agar-Agar, pH 6.5 ± 0.1) [32]. The plates were incubated at 30 °C for five days. After appearing of single colonies, they were picked and transferred into liquid SDA medium to obtain pure cultures. The purity of the cultures was tested by plating again onto solid plates and by PCR (polymerase chain reaction) analysis. *R*. *toruloides* DSM 10134 was obtained from the DSMZ (Leibniz Institute DSMZ-German Collection of Microorganisms and Cell Cultures, Braunschweig, Germany).

### Molecular characterization, amplification of rRNA ITS gene fragments and sequencing

For molecular identification of the isolated microorganisms, cells were grown in liquid SDA medium for 48 h at 30 °C and 130 rpm. 2 mL of the solution were sampled and the DNA was extracted using alkaline lysis [33]. The rRNA ITS gene fragments which are the DNA-barcode established for fungi [34] were amplified using the primer pair ITS1 and ITS4 [35]. PCR was performed as described in White *et al*., 1990. Before sequencing the amplified products, the DNA was purified using the innuPREP-PCR pure Kit (Analytik Jena, Jena, Germany), and eluted in double-distilled water according the instructions of the manufacturer. The purified PCR products were sequenced by GATC (GATC Biotech AG, Konstanz, Germany). The retrieved rRNA ITS sequences were compared with sequences available in the non-redundant nucleotide database of the National Center or Biotechnology database (http://www.ncbi.nlm.nih.gov) using BLASTN and the ribosomal database project (RDP, http://rdp.cme.msu.edu/seqmatch/seqmatch_intro.jsp).

### Heavy metal tolerance (determination of MIC) and use of different carbon sources

For heavy metal tolerance tests with uranium, chromium, zinc, cadmium, and copper, yeast cells (KS5 and DSM 10134) were grown for 48 h in liquid SDA medium at 30 °C and 130 rpm. Subsequently, cells were washed twice in 0.9% NaCl solution to remove the residual ingredient medium. To investigate the tolerance of KS5 and DSM 10134 to heavy metals, the microbial suspensions with an initial OD_600nm_ of 5.0 was 1:500 diluted and 100 μL were plated onto solid agar plates containing SDA 1:5 diluted with adjusted metal concentration (cadmium, copper and chromium 0.1–5.0 mM; zinc and uranium 0.1–10.0 mM), using the nitrate salts of the metals (Cd(NO_3_)_2_ • 4H_2_O, Cu(NO_3_)_2_ • 3H_2_O, Cr(NO_3_)_3_ • 9H_2_O, Zn(NO_3_)_2_ • 6H_2_O, UO_2_(NO_3_)_2_, purity ≥ 98% p.a.). The plates were incubated for 48 h at 30 °C. The minimal inhibitory concentration (MIC) was determined in triplicates. In addition, tolerance test with uranium in liquid medium was determined using the isolate KS5. SDA medium 1:5 diluted was adjusted with different uranium concentrations (0.05, 0.1, 0.2 mM) and was inoculated with 50 μL of a 48 h old culture. After distinct time points, samples were taken to analyze the OD_600nm_. The incubation took place at 30 °C and 130 rpm in triplicates. The resulting growth curves were plotted with logarithmic scale to calculate the growth rate $\mu =\frac{\text{ln}x_{t}-lnt_{0}}{t-t_{0}}$ and doubling time ${\text{t}}_{\text{d}}=\frac{ln2}{\mu }$ within the linear exponential phase. To investigate the use of different carbon sources, KS5 and DSM 10134 were pre-cultured in liquid SDA medium as described above and washed two times with 0.9% NaCl solution. The washed cells were diluted to an OD_600nm_ of 0.1. 50 μL solution from the diluted and washed cell suspension was inoculated in 50 mL minimal-salt-medium (MSM, 0.2 g/L KH_2_PO_4_, 0.6 g/L K_2_HPO_4_, 0.5 g/L (NH_4_)_2_SO_4_, 0.1 g/L MgSO_4_ • 7H_2_O, 0.01 g/L CaCl_2_, 0.005 g/L FeCl_2_, 0.01 g/L MnCl_2_, purity of all reagents ≥ 98%, Merck KGaG, Darmstadt, Germany) which was supplemented with 1% of different carbon sources (lactate, maltose, mannose, fructose, glucose, sucrose, xylose, acetate, oxalic acid, glycerol, ethanol, galactose). The cells were incubated at 30 °C and 130 rpm for 72 h. The experiments were carried out in triplicates of independent biological replicates. Control samples (triplicates) without yeast cells were treated similar as described above. Finally, to investigate the growth with the different carbon sources, the OD_600nm_ was measured.

### Determination of uranium removal capacity

To test the ability to remove uranium from the surrounding solution, yeast cells of KS5 were grown in liquid SDA medium for 48 h at 30 °C and 130 rpm. Afterwards cells were washed three times with sterilized tap water pH 5.0. The washed yeast cells were suspended in the background solution (sterilized tap water pH 5.0) to an initial OD_600nm_ of 1.0 (6.5 mg/mL ± 0.5 mg/mL). Subsequently, uranium as UO_2_(NO_3_)_2_ was added to reach a final concentration of 0.1 mM. To investigate the temperature-dependent uranium removal capacity, the yeast cells were washed with an acclimated background solution at 4 °C or 30 °C. The cell suspensions were incubated at selected temperatures for 48 h. During the incubation, samples were taken at distinct time points (5 min, 0.5 h, 1 h, 1.5 h, 2 h, 4 h, 6 h, 8 h, 26 h, 48 h) to determine the residual uranium concentration within the supernatant. To investigate a possible uranium precipitation, control samples without cells were prepared and simultaneously investigated during all approaches. The samples were centrifuged for 5 min at 13225 x g at RT (Centrifuge 5415R, Eppendorf AG, Hamburg, Germany) and the acidified supernatant (using concentrated, distilled HNO_3_) was analyzed with Inductively Coupled Plasma Mass Spectrometry measurements (ICP-MS) using an ELAN 9000 type ICP-MS spectrometer (Perkin Elmer, Überlingen, Germany). The detection limit of this method is 1μg/L for uranium. To study the uranium removal capacity of dead cells, cultures grown for 48 h were immediately autoclaved for 30 min at 121°C and 1 bar for 30 min. Autoclaved cells were assayed as described above. The amount of removed uranium from the solution was normalized to the dry biomass after drying the cell pellet for 24 h at 100 °C in an oven (Memmert UE500, Schwabach, Germany). To investigate the uranium removal capacity dependent on the initial dry biomass (dbm), different weights of biomass were applied. The cells were incubated with 0.1 mM uranium for 48 h at 30 °C and 130 rpm. Afterwards the cells were harvested and dried as described above. The experiments for all described approaches above were performed by biological independent triplicates.

### Effect of uranium on cellular viability using flow cytometry

To investigate the effect of uranium on the cell, uranium-treated cells were stained with fluorescent dyes and analyzed by flow cytometry techniques. Therefore, an inoculum of a pre-grown culture of KS5 and DSM 10134 was added to 1:5 diluted SDA medium either without uranium as a control or containing a uranium concentration of 0.1 mM. The cells were incubated for 24 h at 30 °C and 130 rpm. Afterwards, the cells were harvested by centrifugation at 7870 x g (Centrifuge 5804R, Eppendorf AG, Hamburg, Germany) for 10 minutes and washed twice with Phosphate Buffered Saline (PBS). Subsequently, the cells were dissolved in PBS to approximately 10^6^ cells/mL. As the ‘dead’ control an aliquot of the cells were incubated for 45 min at 80 °C. The cell viability test was performed with propidium iodide (PI) (stain dead cells) and fluorescein diacetate (FDA) (stain alive cells). The fluorescent dyes were added to a final concentration of 2 μL/mL for PI and 20 μL/mL FDA. The cell suspension was incubated for 15 min in the dark at ambient temperature. After the incubation with the two dyes both strains were analyzed by flow cytometry using a FACSCantoII cytometer Becton Dickinson (San Jose Palo Alto, California) available at the “Centro de Instrumentación Científica” of the University of Granada, equipped with three lasers: 488 nm blue, 620 nm red, and 405 nm violet. All experiments were done in independent biological triplicates.

### Transmission electron microscopy (TEM) and Energy-dispersive X-ray spectroscopy (EDXS)

Immediately after the uranium removal experiments, the cells of three independent replicates were harvested by centrifugation for 10 min at 4427 x g (Centrifuge 5804R, Eppendorf AG, Hamburg, Germany) at room temperature to remove the supernatant. The cell pellet was washed twice with sterilized tap water at pH 5.0 added with 0.2% glucose. Subsequently, the cells were fixed with 2.5% (vol/vol) glutardialdehyde from 50% (vol/vol) stock (Carl Roth, Karlsruhe, Germany) over night at 4 °C. After fixation, the cells were transferred in 4% (w/v) aqueous low-melting agarose (Life Technologies Inc., Darmstadt, Germany), and after cooling, dehydrated by an ethanol series (25, 50, 75, 95% for 10 min; 100% for 2 h; 100% over night at RT), followed by ERL-resin impregnation and polymerization. Sample preparation with minor modifications was done according to the user manual [36]. Ultrathin sections of 100 to 200 nm were cut with a diamond knife (EMS, Munic, Germany) and transferred onto carbon-coated Cu grids (lacey carbon on 200 mesh Cu (S166-2), Plano GmbH, Wetzlar, Germany). TEM investigations were done with an image C_s_-corrected Titan 80–300 microscope (FEI) operated at an accelerating voltage of 300 kV. Qualitative atomic number contrast images were obtained by high-angle annular dark-field scanning transmission electron microscopy (HAADF-STEM). Employing a Li-drifted silicon detector (EDAX) in STEM mode, energy-dispersive X-ray spectroscopy (EDXS) measurements were performed for qualitative chemical analysis. For elemental distribution analysis samples were examined in HAADF-STEM mode with Titan G2 80–300 microscope (FEI) at “Centro de Instrumentatión Cientifica” at the University of Granada, Spain. Prior to each STEM analysis, the specimen holder was plasma-cleaned to minimize contamination.

### Extended X-ray absorption fine structure spectroscopy (EXAFS)

In order to obtain information about the structure of the formed uranium complexes at molecular scale, EXAFS analyses were performed. After uranium immobilization experiments with 0.1 mM uranium, contacted with the yeast cells for 48 h and at 30 °C (described above) the cells of three independent replicates were merged and subsequently ultra-centrifuged (Ultracentrifuge Optima XL100K, Rotor: SW 32Ti; Beckman Coulter, USA) for 1 h at 187000 x g. The supernatant was removed and the resulting cell pellet was placed into polyethylene sample holders. The sample holders were sealed, frozen, and stored in liquid nitrogen until the X-ray absorption measurements. The measurements were carried out at the Rossendorf Beamline BM20 at the European Synchrotron Radiation Facility (ESRF) [37]. The yeast cells were measured at 15 K in a closed-cycle He-cryostat in order to reduce thermal noise and to avoid radiation-induced redox reactions of uranium during the measurements. A water-cooled Si(111) double-crystal monochromator in channel cut mode (5–35 keV) was used to monochromatize the incoming synchrotron X-rays. In dependence of the uranium amount the spectra were collected in fluorescence mode or in transmission mode using ionization chambers. A reference sample, meta-autunite Ca(UO_2_)_2_(PO_4_)_2_ • 6H_2_O [38], was measured at room temperature in transmission mode [39]. The K-edge spectrum of an yttrium metal foil (first inflection point at 17038 eV) was recorded simultaneously for energy calibration of the sample spectra. *E*_*0*_, the ionization energy, of the uranium L_III_-edge was defined as the maximum of the second derivative of the averaged spectra. Eight scans in fluorescence mode were collected of the yeast cells incubated with 0.1 mM uranium. The fluorescence spectra were corrected for the detector dead time and subsequently averaged. The spectra were analyzed using the data analysis programs Sixpack/SamView (Version 0.59) (Webb 2005) and WinXAS (version 3.11) [40].

## Results and discussion

### Phylogenetic affiliation, morphological characterization and utilization of different carbon sources of KS5

Culture dependent methods based on the use of SDA medium resulted in the isolation of different microbial strains. SDA medium was developed and used for enrichment of fungal stains from environmental and clinical samples [32]. Single colonies obtained were used for the enrichment of pure cultures indigenous within the flooding water of Königstein. On SDA agar medium, the isolate KS5 displayed red round and shiny colonies (S1 Fig). The phylogenetic affiliation of the microbial isolate based on ITS rRNA gene analysis displayed high similarity to *R*. *toruloides* (strain JZ-9, 99% identity and 100% query cover). These yeast cells are known for their production of lipid related molecules, including biodiesel, adhesives, and high-value nutritional oils [41–47]. In addition, *R*. *toruloides* is able to utilize a wide variety of carbohydrates derived from plant biomass, including xylose, and cellobiose [47–49]. To investigate the isolated strain in more detail, studies on the metabolic versatility regarding the use of different carbon sources were performed. In addition, the reference strain DSM 10134 was investigated in the same way. These experiments were performed in order to find a suitable carbon source to grow the isolated strain KS5 directly within the flooding water for *in situ* bioremediation approaches. The results (Table 1) displayed slight differences for the carbon sources mannose, acetate and ethanol. KS5 displays better growth on mannose and acetate, compared to DSM 10134. Notably, KS5 shows the ability to grow in the presence of xylose, in contrast to DSM 10134 which was not able to metabolize this sugar. The fermentation of xylose to ethanol by yeasts was well studied by several investigations and could be a useful process for the production of bioethanol [50–53]. Nevertheless, the results show that the uranium bioremediation potential of the isolated strain KS5 could be enhanced by the ability to use different sugars like maltose, fructose, mannose and sucrose.

**Table 1:** Growth on different carbon sources of KS5 and DSM 10134.

Carbon source	KS5	DSM 10134
**lactate**	**+**	**+**
**maltose**	**++**	**++**
**mannose**	**++**	**+**
**fructose**	**++**	**++**
**glucose**	**+**	**+**
**sucrose**	**++**	**++**
**xylose**	**+/-**	**-**
**acetate**	**+**	**+/-**
**oxalic acid**	**-**	**-**
**glycerol**	**+**	**+**
**ethanol**	**+/-**	**+**
**galactose**	**+/-**	**+/-**

The two strains were grown in liquid mineral-salt-medium which was added with 1% carbon source. Growth was determined by measuring of the OD_600nm_. ++ good growth (OD > 0.4), + growth (OD 0.2–0.39), +/- less growth (OD < 0.2),—no growth (OD = 0.0) (n = 3).

### Impact of uranium and selected heavy metals on microbial growth

**Fig 1:**
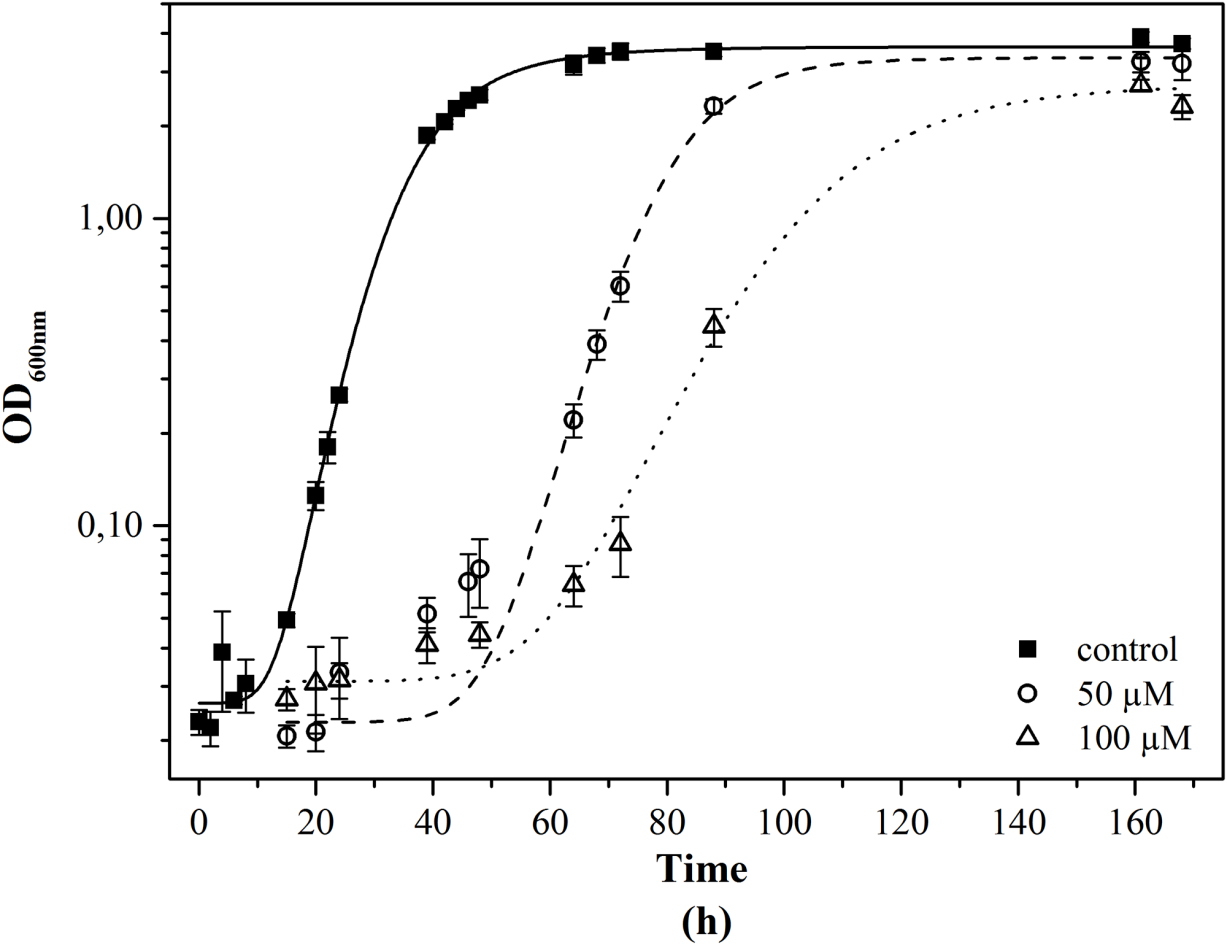
Growth curves of KS5 incubated with different uranium concentrations, the cells were grown in liquid SDA 1:5, uranium was added in different concentrations, squares no uranium, circles 0.05 mM uranium and triangle 0.1 mM uranium, incubation for 172 h at 30 °C (n = 3 for each approach, error bars indicate the standard deviation).

To investigate the impact of uranium on the microbial growth, 1:5 diluted SDA medium with increasing metal concentrations was used. The growth curves of KS5 incubated with two different uranium concentrations are displayed in Fig 1. It is clearly visible that the curves with uranium (0.05 and 0.1 mM) are clearly shifted compared to the control (without uranium). The lag-phase was longer which might originate from a possible adaption of the cells to uranium. Based on the growth curves, we calculated the growth rate μ and doubling time t_d_ for all three approaches (Table 2). Compared to the uranium-free control, a decrease of the growth rate μ with increasing metal concentrations was observed. At the highest uranium concentration of 0.1 mM, the growth rate is more than half times smaller compared to that of the control. Furthermore, the doubling times increased with increasing uranium concentration as well. At 0.1 mM, the doubling time is more than twice higher. Nevertheless, the yeast cells are able to grow up to a uranium concentration of 0.1 mM in liquid SDA medium.

**Table 2:** Growth rate and doubling time of KS5 growing with different uranium concentrations.

	Control	0.05 mM	0.1 mM
**Growth rate μ [h**^**-1**^**]**	0.19	0.13	0.08
**Doubling time t**_**d**_ **[h]**	3.73	5.55	8.56

The cells were grown in liquid SDA 1:5, uranium was added in different concentrations (0.05 and 0.1 mM), incubation for 172 h at 30 °C (n = 3).

**Fig 2:**
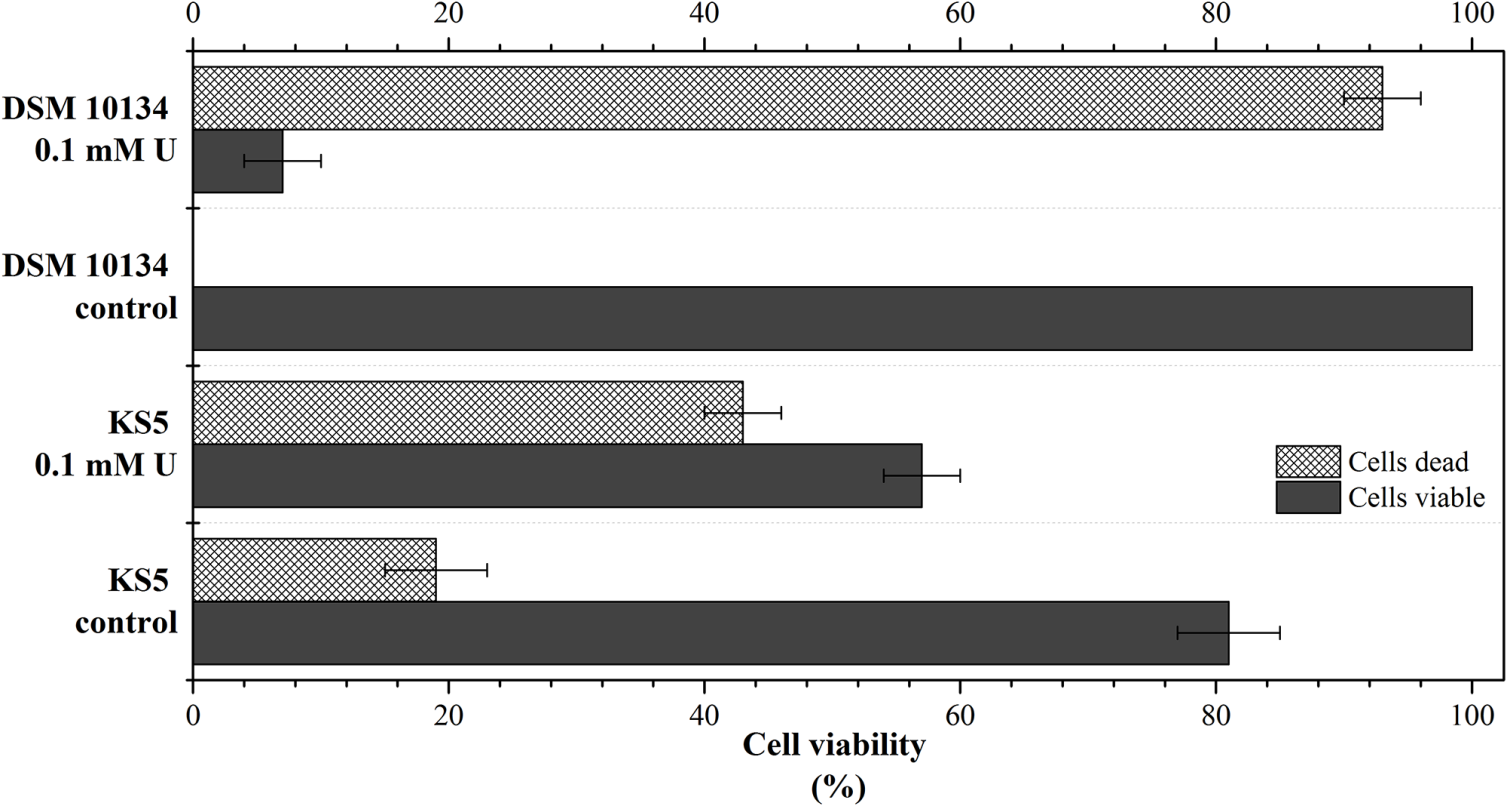
Percentage distribution of viable and dead cells using flow cytometry, cells of KS5 and DSM 10134 were incubated with 0.1 mM uranium or without (control) in 1:5 diluted SDA medium for 24 h at 30 °C. Cells were stained with FDA and PI for cell viability test. Viable (living cells) are displayed in dark grey and dead cells in grey pattern (n = 3 for each approach, error bars indicate the standard deviation).

Furthermore, the uranium tolerance of KS5 in comparison to the reference strain DSM 10134 was studied using flow cytometry. The cells were grown in 1:5 diluted SDA medium supplemented with 0.1 mM uranium. After an incubation time of 24 h, the cells were stained with PI and FDA. The percentage distribution of viable and dead cells with and without uranium is displayed in Fig 2. In the absence of uranium, more than 80% of the KS5 and all the DSM 10134 cells displayed viability. At a uranium concentration of 0.1 mM, the two microbial strains tolerate this metal in different ways. The cell viability was reduced to 60% and 10% for KS5 and DSM 10134, respectively. The high uranium tolerance of KS5 could be explained by adaption of this strain to uranium in its natural habitat contaminated with uranium. To survive within the flooding water, containing high amounts of uranium, KS5 has to develop adaption mechanisms. Previous studies have shown that microorganisms, which were isolated from contaminated sites, display the ability to tolerate relatively higher concentrations of heavy metals [54–56]. These natural occurring microbes could be promising candidates for their use in the bioremediation of these inorganic contaminants [31]. In addition, Sakamoto *et al*., 2012 [57] concluded that several genes of *S*. *cerevisiae* are involved in uranium tolerance. Phosphate transporter genes were observed to be responsible to contribute to uranium tolerance and furthermore, cell surface proteins contributed to the uranium accumulation [57]. Furthermore, experiments on the tolerance of *S*. *cerevisiae* toward uranium revealed high survival rates at tremendous high uranium concentrations. Over an exposure time of 8 days nearly 20% of the cells survived at a uranium concentration of 1.7 mM (400 mg/L) [58]. Although KS5 displayed a lower uranium tolerance, further investigations should be performed to investigate the survival rate over a longer incubation time. However, additional investigations with the isolated strain KS5 should be performed to identify possible gene responsible for the high uranium tolerance.

To evaluate the tolerance against selected heavy metals (Cr, Cu, Cd, Zn) and uranium. KS5 was grown on solid agar plates with increasing concentrations of metals. To compare possible adaption mechanisms of the indigenous yeast KS5 the reference strain, DSM 10134, was investigated in the same way. The results of the heavy metal tolerances (Table 3) showed clear differences between the two strains. The investigated uranium tolerance on solid agar plates revealed a six-fold higher tolerance of the natural isolate (6.0 mM) compared to the reference strain (1.0 mM). Previous studies using other isolated yeasts from Spanish clay deposit showed similar results. Two isolated strains from bentonite samples exhibit tolerances up to 10 mM for uranium [59]. In addition, also for chromium KS5 showed a distinct higher tolerance than the reference DSM 10134. An isolated strain of *Rhodosporidium* sp. from metallurgical wastes displayed a similar high resistance to chromium [60]. However, for copper, cadmium and zinc, we couldn´t see obvious differences between the natural isolate and the reference strain.

**Table 3:** MIC (mM) for KS5 and DSM 10134 on SDA medium 1:5 diluted.

	Uranium	Chromium	Copper	Cadmium	Zinc
**KS5**	6.0	5.0	0.4	< 0.1	1.0
**DSM 10134**	1.0	1.0	0.3	< 0.1	0.7

MIC: concentration at which no growth was occurred (n = 3).

Our investigations on uranium and selected heavy metals tolerance revealed a possible evolutionary adaption of KS5 to the surrounding environmental conditions. Compared to the reference strain, DMS 10134, the indigenous yeast exhibits tremendously high tolerances of uranium and chromium. To identify the molecular mechanisms behind, these high tolerances gene expression analysis should be performed. Nevertheless, the results display the ability of KS5 to survive high concentrations of uranium as well as other heavy metals and thus, this indigenous yeast could play an important role of *in situ* bioremediation approaches of contaminated sites.

### Uranium removal capacity of KS5

In order to determine the influence of KS5 on the immobilization capacity of uranium at natural conditions, uranium removal studies were performed at pH 5.0. Several previous studies demonstrate that the optimum pH value for uranium immobilization by fungal biomass is between 4.0 and 6.0 [61–63]. Due to the relation of biosorption to the number of negative surface groups, which is depending on the dissociation of these functional groups, this phenomenon could be explained. The majority of the functional groups at microbial membranes become dissociated only at neutral pH values, thus only a low amount of metal ions retained at microbial sorbents at pH values below 4.0 [62]. Furthermore, at pH 5.0 uranium was predicted (data not shown) to be soluble at the prevalent conditions within the tap water. The control samples without microbial cells confirmed this, due to the reason that no precipitation of uranium occurred.

**Fig 3:**
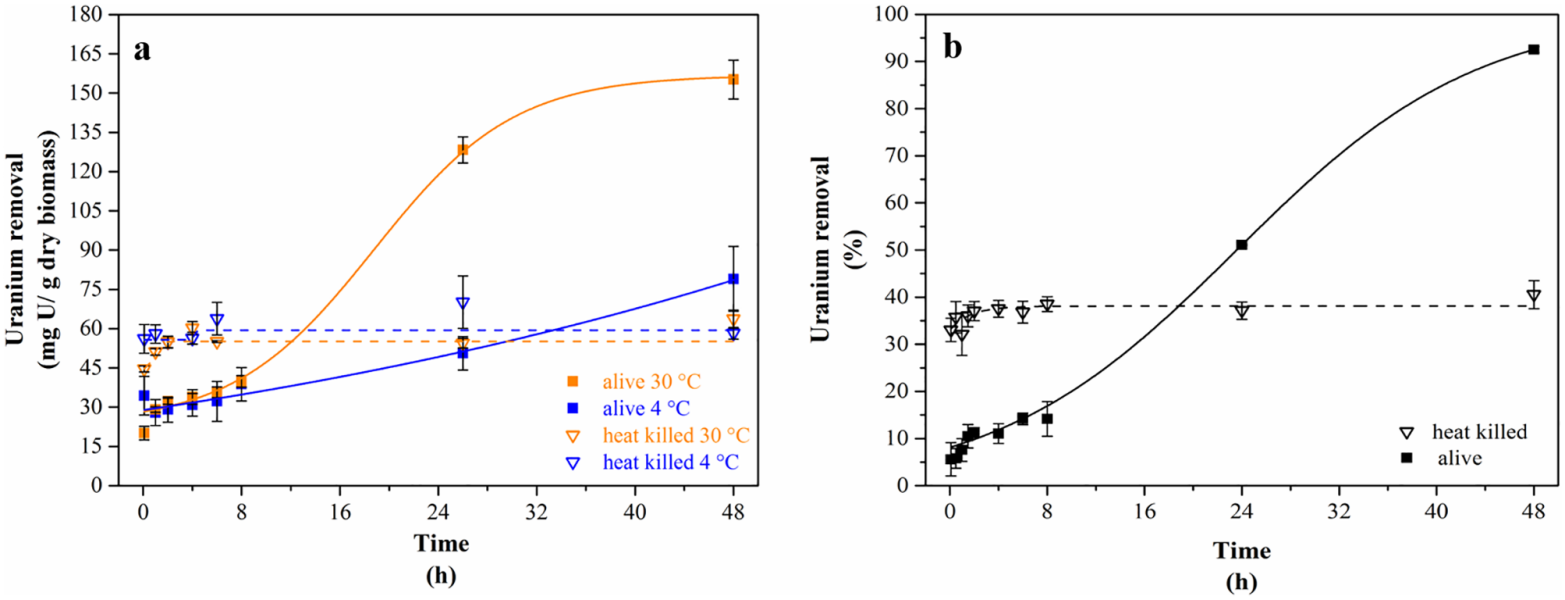
Temperature-dependent uranium removal capacity of KS5 (a) Uranium removal relating to dry biomass, orange curves show incubations at 30 °C and blue curves at 4 °C, filled scares display living cells (solid lines) and open triangles display heat killed cells (dashed lines); (b) Percentage uranium removal of living cell (solid line and filled scares) and heat killed cells (dashed line and open triangles) at 30 °C; initial uranium concentration 0.1 mM; background solution tap water pH 5.0 (n = 3 for each approach, error bars indicate the standard deviation).

The investigations on uranium removal by cells of the strain KS5 displayed that uranium was removed rapidly from the surrounding solution within 24 h. After 48 h incubation, metal binding saturation by the cells was reached. The uranium removal capacity of the strain KS5 is a temperature-dependent process as was indicated in Fig 3a. The cells were able to remove around 150 mg U/ g dry biomass (dbm) from the surrounding solution at 30 °C, whereas at 4 °C, the cells removed only around 75 mg U/ g dbm. Experiments with heat-killed cells incubated at the same temperatures revealed that dead cells remove lower amounts of uranium compared to living yeast cells. Nevertheless, the amount of removed uranium of around 60 mg U/ g dbm is almost equal to the amount for cells incubated at 4 °C. Furthermore, regarding to the percentage removal of uranium (Fig 3b), living cells of KS5 removed nearly 100% of soluble uranium from solution. In contrast, only 40% of uranium was removed by heat killed cells. These findings imply that uranium removal by KS5 cells is metabolism-dependent. Moreover, the temperature-dependent experiments indicate that the uranium removal is based on different interaction processes, probably passive biosorption and active bioaccumulation. In addition, the less amount of removed uranium by dead cells of KS5 could prove the occurrence of metabolically-dependent processes. The process of biosorption is rapid and will be not affected by temperature due to the metabolism-independent sorption of uranium on negatively charged groups of the cell membrane [64,65]. In contrast, the mechanism of intracellular bioaccumulation of actinides especially uranium is poorly understood. However, previous studies assumed an active transport of uranium into the cells [66]. The same temperature-dependent and thus possibly metabolism-dependent process was observed by uranium interaction experiments on *A*. *facilis* [67]. Similar to KS5, the bacterial cells removed lower amounts of uranium at lower temperatures.

Compared to other yeast cells, like *S*. *cerevisiae*, KS5 could remove distinct higher amounts of uranium from the surrounding solution [68]. Although the authors used lower concentrations of the metal, the studied strain removed only 40% of the initial concentration, which correspondents to a uranium removal capacity of 17 mg U/ g dbm. In addition, they investigated the removal capacity of living and dead cells for comparison purpose. On the contrary to our findings *S*. *cerevisiae* accumulated higher amounts of uranium onto dead cells [68]. Indicating again that active mechanisms, such as bioaccumulation, could be responsible for the uranium removal by living cells of KS5 further to the passive process of biosorption. STEM analyses (results discussed below) support this assumption.

**Fig 4:**
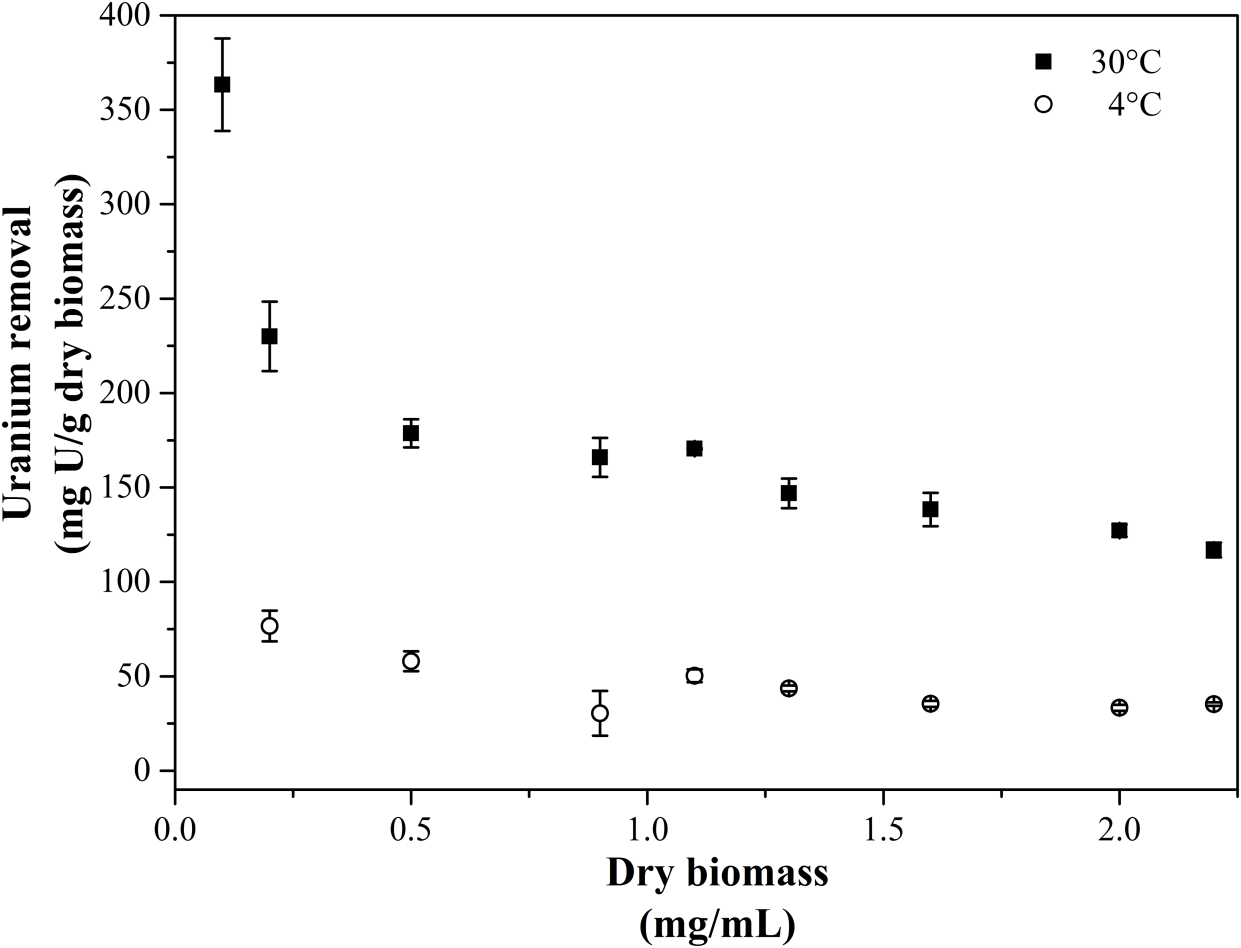
Uranium removal capacity by KS5 as function of the initial dry biomass, initial uranium concentration 0.1 mM, background tap water pH 5.0, incubation time 48 h at 30 °C (filled squares) and 4 °C (open circles) (n = 3, error bars indicate the standard deviation).

Fig 4 shows the effect of biomass concentration of KS5, ranging from 0.05 to 0.24 mg/mL on uranium removal capacity. The results reveal that uranium binding the capacity of the isolate KS5 decreased with the increase of biomass concentration from 0.05 to 0.1 mg/mL, accumulating up to 350 and 175 mg U/ dbm, respectively. Afterwards, the equilibrium of uranium removal capacity was reached at around 150 mg U/g dbm and almost the complete amount of dissolved uranium was removed by the yeast cells. In addition, the removal capacity dependent on dry biomass displays the same result as the kinetic studies mentioned above. The ability to remove uranium was different at two tested temperatures. Cells, which were incubated at 4 °C removed much less uranium compared to cells at 30 °C. Even at the lowest initial dry biomass only around 80 mg U/ g dbm was removed by the yeast cells. Compared with other fungal strains, KS5 displays a high capacity of uranium removal. All observed *Rhizopus* strains displayed a capacity between 180 and 260 mg U/ g dbm [69]. In contrast to bacterial cell such as *Paenibacillus* sp. JG-TB8, which was recovered from a soil sample of another uranium mining site (Johanngeorgenstadt, Germany) and displayed a uranium removal capacity of 138 mg U/ g dbm (at pH 4.5) [70], KS5 shows a higher capacity. Compared to the model organism *S*. *cerevisiae*, which displayed maximum biosorption quantity of 102 mg uranium/ g dbm [71], KS5 was able to remove more than twice. The biosorption of heavy metals especially uranium by yeast cells was shown in previous studies and confirmed our investigations of actively intracellular uptake [72,73].

The results of the uranium removal capacity by the isolated strain KS5 demonstrated its fast and efficient immobilization. Upscaling of the here presented experiments, might verify possible *in situ* bioremediation approaches. However, by using an indigenous strain, additional processes to produce high amounts of biomass would unnecessary, due to the reason that KS5 could be grown directly within the flooding water. In further steps the recovery of the uranium removed by the cells should be taken into consideration. Previous studies revealed that the subsequent treatment with EDTA leads to a desorption of uranium from the microbial cells [74]. Nevertheless, it has to be mentioned that desorption processes are only possible at the interaction mechanism of biosorption. For industrial applications by using the isolate strain KS5 for bioremediation approaches further studies should be performed to prove our findings and to support or even replace the conventional waste water treatment at the site of the former uranium mine Königstein.

### Localization of removed uranium by TEM studies

**Fig 5:**
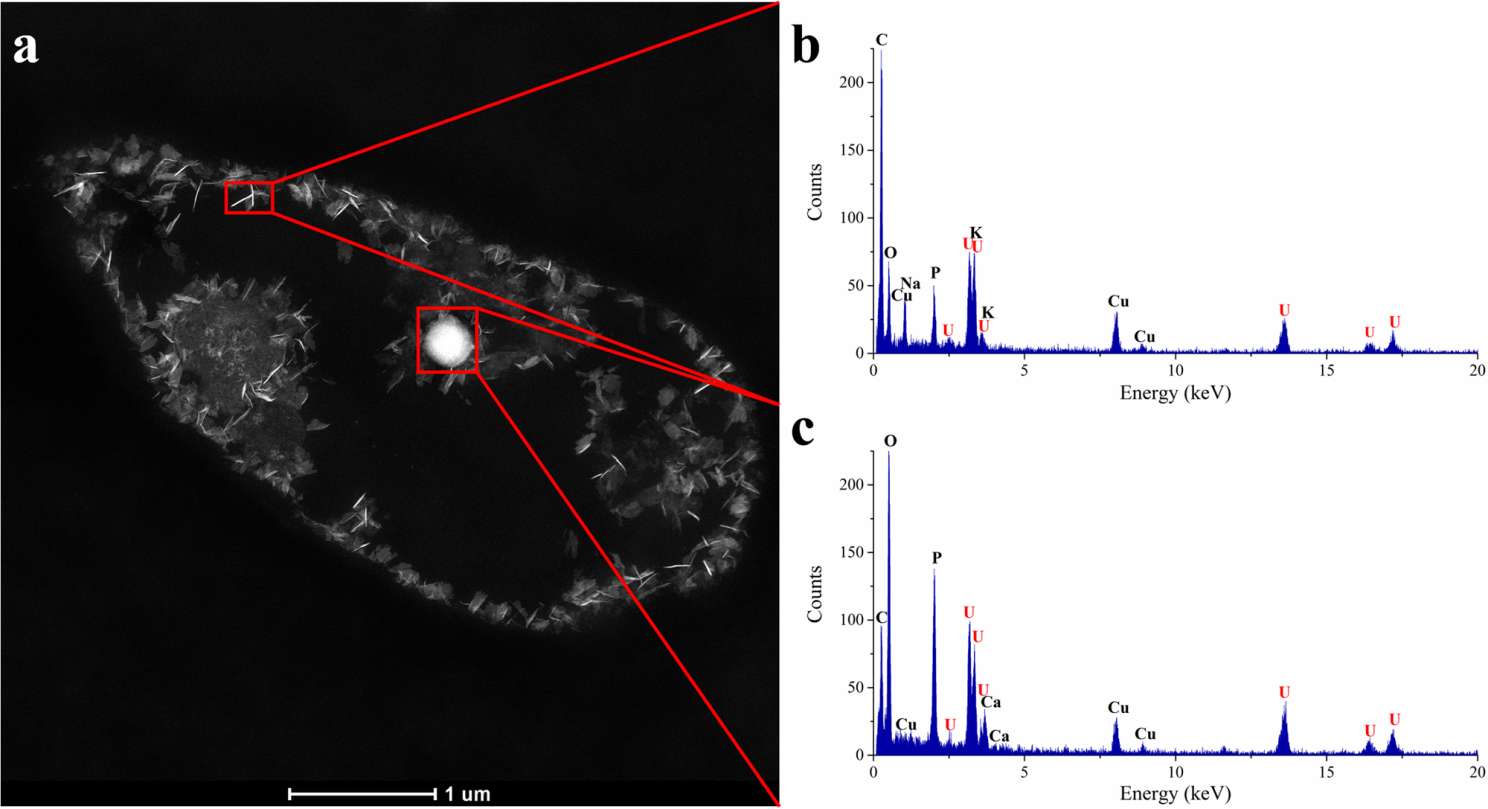
STEM-EDXS analysis of an ultrathin-sectioned KS5 sample after uranium removal experiments at 30 °C for 48 h (a) HAADF-STEM micrograph together with (b) EDX spectra of a needle-like structure localized at the inner cytoplasm-membrane, and (c) of immobilized uranium localized within lipid granules. The characteristic peaks of copper in the EDX spectra are caused by fluorescence excitation of the TEM support grid.

**Fig 6:**
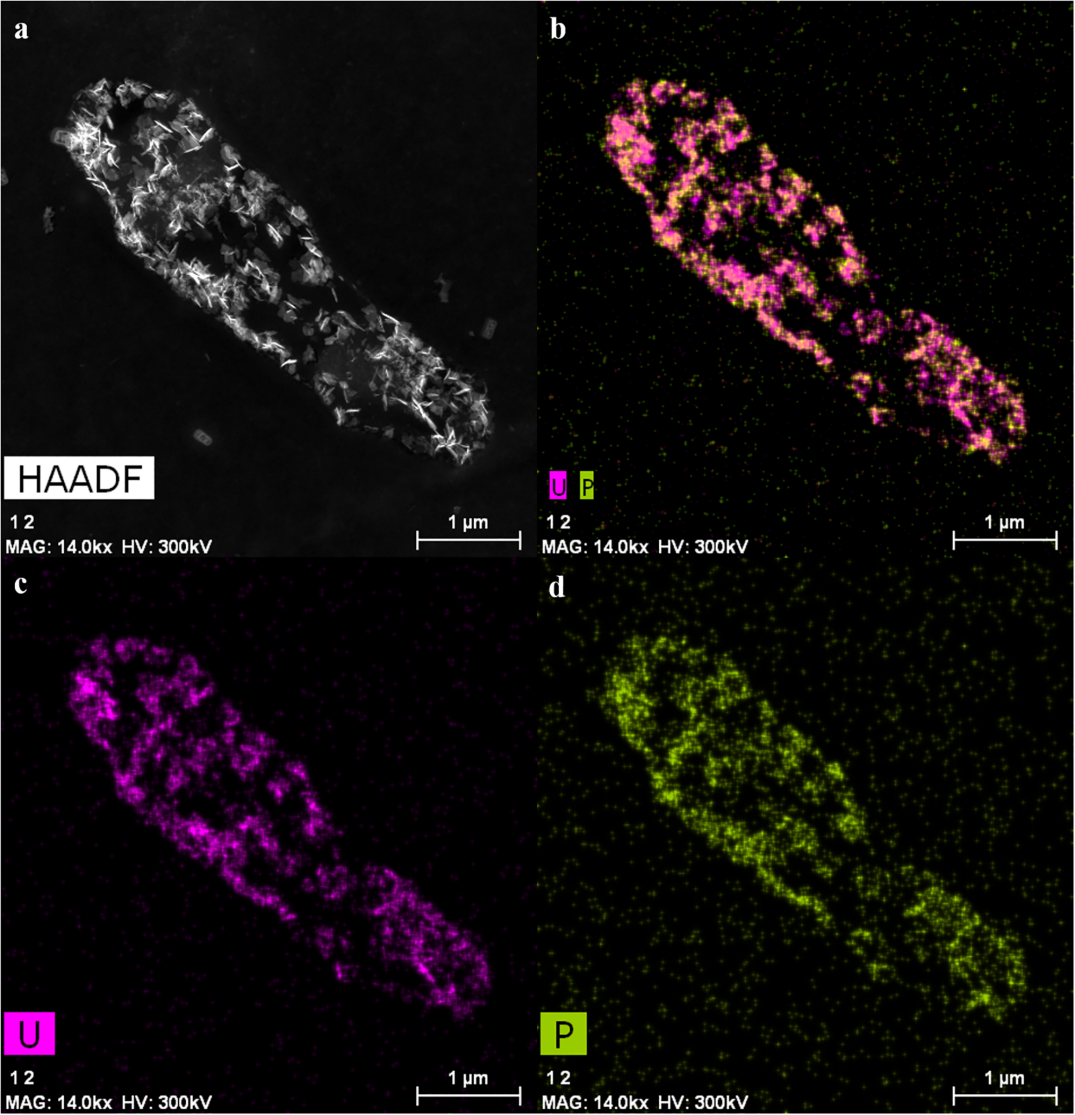
HAADF-STEM analysis of an ultrathin-sectioned KS5 sample after uranium removal experiments at 30 °C for 48 h (a) HAADF-STEM micrograph together with distribution analysis of (b) uranium (purple) and phosphorus (green), (c) uranium, and (d) phosphorus.

Transmission electron microscopy analyses were performed to investigate the cellular localization of uranium accumulated by the cells of KS5, and to elucidate the possible uranium interaction mechanisms with this yeast. The temperature-dependent uranium removal capacity tests, conducted at 4 °C and 30 °C, suggest the implication of two possible interaction mechanisms, namely passive biosorption and active bioaccumulation. Fig 5a shows a STEM image of KS5 incubated at 30 °C together with two EDX spectra obtained for two regions of metal accumulates (Fig 5b and 5c) localized within the cells. Intracellular uranium is detected in the form of phosphorous-containing needle-like structures which are localized at the plasmatic membrane, as well as at the outer membrane of the nucleolus. Additionally, uranium is associated in lipid granules localized within the cytoplasm. Several studies showed that *R*. *toruloides* is known for overproduction of lipids and pigments [44,75–77] and for the formation of lipid droplets which serve as energy reservoir [78]. To investigate the possible binding sites of uranium, further element distribution analyses were performed (Fig 6a–6d). The results clearly indicated the common presence of uranium together with phosphorus (needle-like structures) (Fig 6b).

In contrast, when cells incubated at 4 °C (S2 Fig), it is clearly visible that, considerably less uranium amount was immobilized by the cells. Uranium is localized only at the outer membrane and is not taken up into the cell. Due to the fact, that only cells which were incubated at 30 °C display intracellular accumulated uranium whereas cells incubated at 4 °C show no uranium inside the cells, it could be assumed that uranium is actively accumulated.

In comparison to other yeast strains, the same needle-like fibrils were observed [68]. In contrast to our results, however, the authors could not detect any uranium accumulates inside the yeast cells. Furthermore, Strandberg et al. 1981 [79] suggested no metabolisms-dependent uranium interaction mechanism by *S*. *cerevisiae* which seems to be in disagreement with the finding reported in the present work, where the cells of the strain KS5 interact with uranium in a metabolisms-dependent way due to the temperature-dependent uranium immobilization capacity. Other studies on naturally isolated yeast cells from Spanish clay deposits confirmed a similar uranium immobilization behavior [59]. Uranium was precipitated on the outer cell surface as well as intracellularly. In addition, also needle-like structures of the immobilized uranium could be observed and were identified as uranyl-phosphate minerals with a structure similar to H-autunite [59]. Resulting from the TEM investigations, we confirmed our previous results and our hypothesis that uranium was immobilized by two different mechanisms, by active bioaccumulation and by passive biosorption.

### Speciation of removed uranium by EXAFS analysis

**Fig 7:**
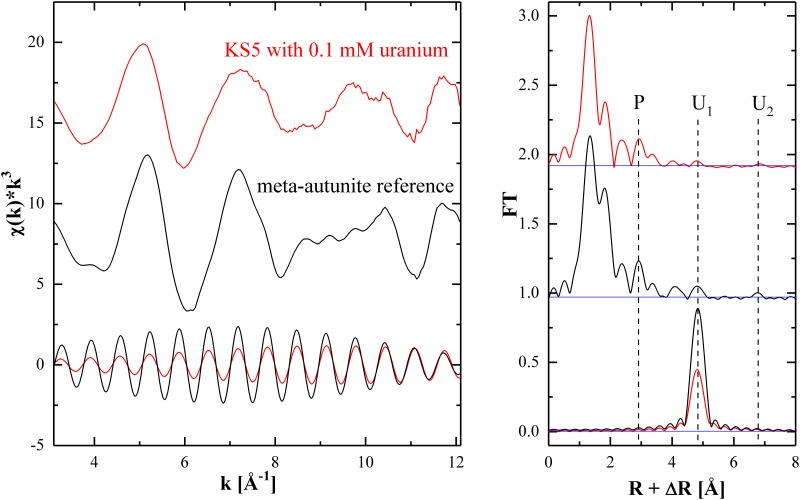
UL_III_ EXAFS spectra (left) and corresponding Fourier transform (FT) (right), KS5 was incubated with 0.1 mM uranium at 30 °C for 48 h (shown in red), the reference meta-autunite (shown in black) together with the Fourier filtered uranium interaction at 4.8 Å (bottom). The noise level (background) was determined from the FT peak magnitude in the 15–20 Å R-region, where no significant signal from the sample itself is expected (blue line).

EXAFS measurements were performed to determine and identify the structure of uranium immobilized by the isolated strain KS5. Fig 7 displays the k^3^-weighted EXAFS spectra and their corresponding Fourier transforms (FT) of the yeast cells contacted with 0.1 mM uranium at 30 °C for 48 h and of the meta-autunite reference. The FT signal of the uranium (U) interaction (Fig 7, U_1_) at 4.8 Å (not corrected for phase shift) was Fourier filtered in the R-interval of 4.53 Å–5.18 Å for both samples. When comparing the signatures in the FT of the uranium solid formed upon contact with the KS5 strain (red traces) with the reference material meta-autunite (black traces), strong similarities can be seen, pointing toward the formation of a meta-autunite-like biomineral under the influence of the yeast cells. A strong indication of meta-autunite formation as a consequence of the biomineralization processes is the detected U-P interaction at R+ΔR = 2.9 Å and the U-U interactions at R+ΔR = 4.8 Å (U_1_) and at R+ΔR = 6.8 Å (U_2_) (Fig 7). Moreover, the Fourier filtered U_1_ signals are in phase (Fig 7, left), showing that the radial U-U_1_ distance is the same for both samples. Though, the presence of other uranium species in minor contributions cannot completely be excluded, since the spectrum of the yeast sample does not match exactly with that of meta-autunite. According to the TEM studies, uranium was mainly removed by bioaccumulation within the cytoplasm and bonded via protonated phosphoryl containing groups. The formation of meta-autunite, as a response of uranium interaction with microorganisms, was mentioned by few studies [19,80,81]. However, no study with *R*. *toruloides* and meta-autunite formation is known. The removal of uranium by yeast cells and its resulting immobilization by formation of uranium minerals may play an important role in bioremediation of uranium contaminated sites due to their stability for long time periods [82].

## Conclusion

Our present study describes the interaction mechanisms of KS5 with uranium(VI) and its tolerance to selected heavy metals. Uranium removal studies and TEM analyses revealed that the cells of the strain interact with uranium through a temperature-dependent process. For yeast cells incubated at 30 °C, intracellular uranium accumulates as needle-like structures were detected in the cytoplasm and also within lipid-granules, which might be a consequence of different detoxification mechanisms. Our findings confirm, that natural occurring microorganisms may play an important role in predicting the transport and fate of uranium at contaminated sites which could be used for *in situ* bioremediation. Moreover, our findings demonstrate that the yeast cells are efficient in the uranium removal from surrounding solutions and could be an alternative for further bioremediation approaches or used as supporting processes in remediation processes at uranium-contaminated sites. However, additional investigations should be performed to examine and confirm the uranium removal capacity of KS5 in industrial scale application. Moreover, investigations should be performed on uranium recovery from the yeast cells.

## Supporting information

S1 Fig Colonies of KS5. Red colonies of KS5 appearing on solid SDA agar plates, incubation at 30°C for 48 hours.
(TIF)

S2 Fig STEM-EDXS analysis of an ultrathin-sectioned KS5 sample. After uranium removal experiments at 4°C for 48 h (a) HAADF-STEM micrograph together with EDX spectra (b) of a needle-like structure localized at the outer cytoplasm membrane.
(TIF)
