# Supplementary figures and images for: Metabolism-dependent bioaccumulation of uranium by Rhodosporidium toruloides isolated from the flooding water of a former uranium mine
Source: PLoS One. 2018 Aug 8;13(8):e0201903. doi: 10.1371/journal.pone.0201903 (PMC6082562; doi:10.1371/journal.pone.0201903)

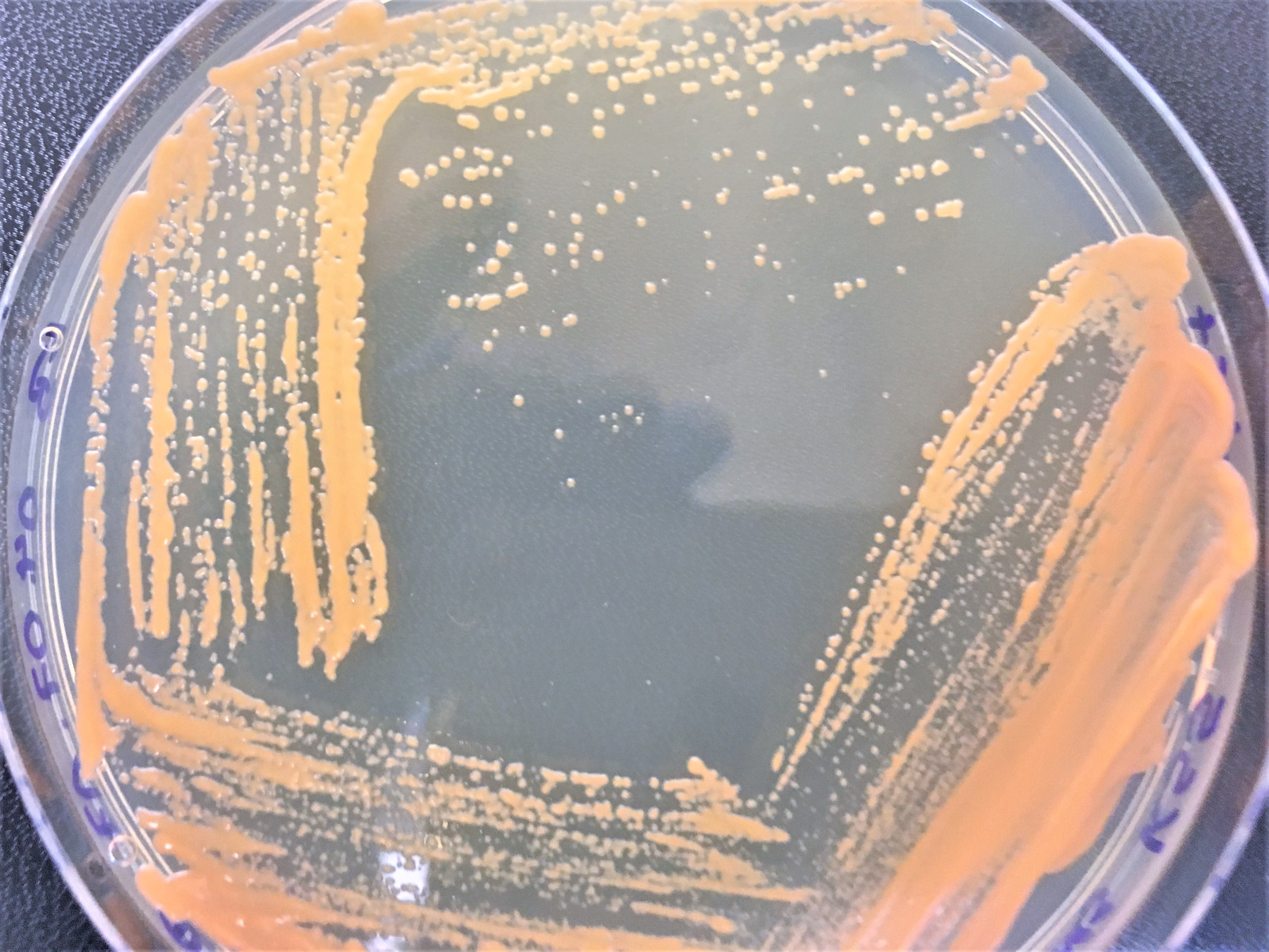

Supplement: S1 Fig — Red colonies of KS5 appearing on solid SDA agar plates, incubation at 30°C for 48 hours. (TIF) [file pone.0201903.s001.tif]

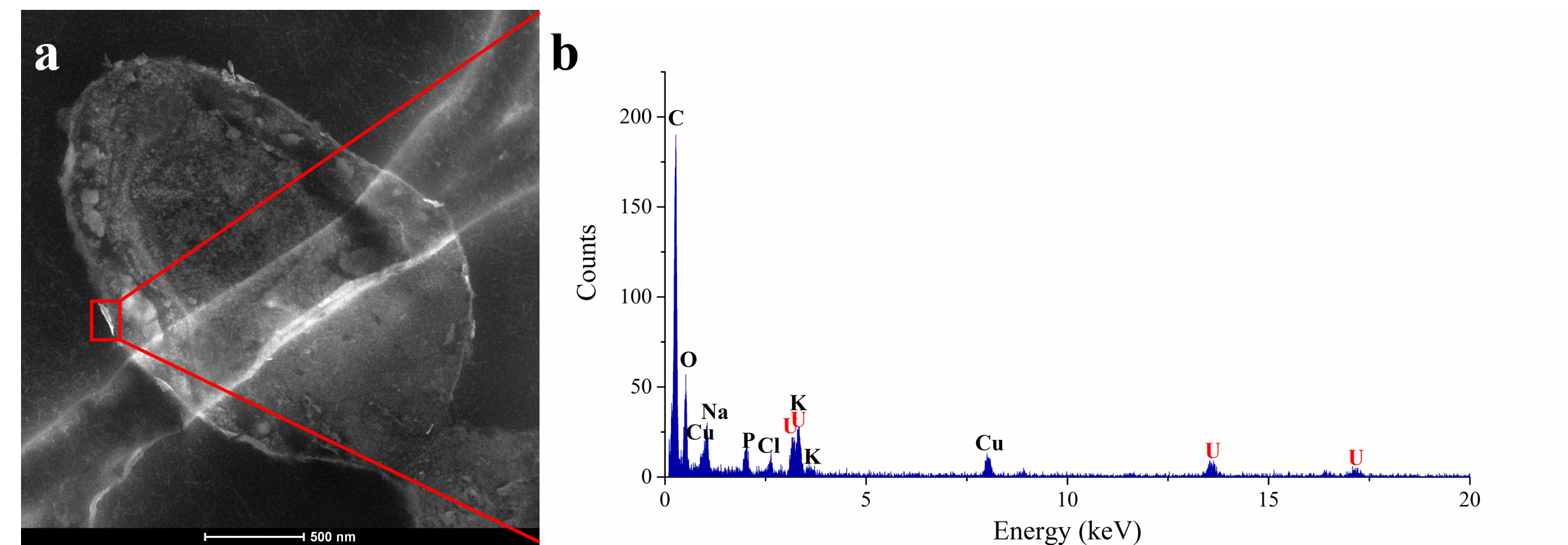

Supplement: S2 Fig — After uranium removal experiments at 4°C for 48 h (a) HAADF-STEM micrograph together with EDX spectra (b) of a needle-like structure localized at the outer cytoplasm membrane. (TIF) [file pone.0201903.s002.tif]
